# Smoking Cessation Level of Contemplation in the Pediatric Emergency Department

**DOI:** 10.19080/ajpn.2018.07.555761

**Published:** 2018-07-26

**Authors:** K Arzubi Hughes Michelle, Mahabee-Gittens E Melinda, Dowd M Denise, Giovanni Joan

**Affiliations:** Department of Pediatrics, Children’s Hospital of The Kings Daughter, USA

**Keywords:** Smoking, Pediatric, Cessation level, Tobacco, Health risk, Correlated

## Background

Smoking tobacco is a significant health risk not only to the person smoking, but to those individuals that are exposed to the second-hand smoke as well. Although the number of smokers in the United States is declining, the effects of tobacco are still abundant and seen alarmingly in children. In the United States, there is an estimated 46 million people or 20.6% of all adults (aged 18 years and older) who smoke cigarettes. Cigarette smoking is more common among men (23.1%) than women (18.3%). These statistics split into age ranges are as follows: 21.4% of adults aged 18–24 years, 23.7% of adults aged 25–44 years, 22.6% of adults aged 45–64 years and 9.3% of adults aged 65 years and older. Most importantly, 43% of children < 12 years old live with at least one smoker [1].

The risks of second and third hand smoke on children’s health are vast. Published studies have correlated the effects of second and third hand smoke to Sudden Infant Death Syndrome (SIDS), respiratory tract infections, asthma, otitis media, behavior problems and sleeping disorders [2]. Environmental tobacco smoke exposure is the leading cause of childhood morbidity and mortality and has been responsible for $4.6 billion in direct medical costs to the treatment of children [3].

It is important to understand the level of contemplation and motivation of smokers to assist physicians in implementing a smoking cessation program and intervention for the particular individual.

The Emergency Department (ED) has been noted to be an ideal location for intervention. Several surveys show that the ED is a point of access to reach large number of smokers [4]. One study concluded that approximately 50% of smokers reported moderate interest in an ED - based intervention and were willing to stay an extra 15minutes to obtain further education [5].

Possible Study Implications: This study will illustrate the relationship between parent’s level of contemplation to quit smoking with their knowledge of smoking and the effects of second hand smoke on their children. By understanding this relationship, adequate interventions such as education and smoking cessation information can be developed for these parents in the Pediatric Emergency Department. Interventions based on the stages of change model have been shown to enhance motivation and predict cessation [6].

## Study Aims

### For smoking parents/primary caregivers of children presenting the ED/UC

- To determine level of contemplation/motivation of parents/primary caregivers to quit smoking.
- The “stages of change” model will be used for assessing a patient’s willingness to quit using tobacco. This model entails changing behavior through a process of five motivational stages:
  - Precontemplation (not planning to quit within the next six months)
  - Contemplation (considering quitting within the next six months)
  - Preparation (planning to quit within the next 30 days)
  - Action (successfully quitting for less than six months), and
  - Maintenance (successfully quitting for at least six months).
- To determine level of understanding of the relationship of smoking to their child’s illness, and childhood smoking-related illnesses in general.
- This will be based on their knowledge of effects of second hand smoke and their child’s chief complaint.
- To measure the association between #1 and #2.
- To determine the inter-relationship between the importance of smoking cessation and #3.

## Methods

### Definitions

- **Level of contemplation:** will be determined by questions #16-#18 (see attached document) in a binary method. Precontemplation: #16 Yes (1), #17 No (0), #18 No (0); Contemplation: #16 Yes (1), #17 Yes (1), #18 No (0); Preparation: #16 Yes (1), #17 Yes (1), #18 Yes (1).
- **Level of knowledge of the effects of second hand smoke:** will be based on question #24. The answers will be dichotomized. The answers that are correct will be given 1 point; those that are incorrect will be given 0 points. The points will then be summed, to a maximum score of 9 points. This score will then be compared in a chart form to the three different levels of contemplation.
- **Correlation of child’s illness:** The child’s diagnosis will be obtained and recorded prior to the discharge order given by the attending.
- **Definition of importance of smoking cessation:** will be based on question #15.

### Study Type

Cross sectional anonymous adult survey of 200 parents will be administered by a research assistant, a small group of sub-investigators or Dr. Michelle Arzubi. A waiver for informed consent will be requested and, in its place, a handout explaining the study will be given to the parents prior to the onset of the study.

### Study site

Emergency Department and Urgent Care of Children’s Mercy Hospital (CMH) Main Campus, Urgent Care of CMH South and North.

### Inclusion criteria

- Parents/primary caregivers of children presenting to the Children’s Mercy Emergency Department, Children’s Mercy Northland Urgent Care, or Children’s Mercy South Urgent Care with non-critical complaints.
- Live in the same household with child.
- English or Spanish speaking.
- Smokers.
- An adult, who has smoked at least 100 cigarettes in his or her lifetime, is currently smoking either inside or outside of the house and may or may not smoke every day.

### Exclusion criteria

- Parents/primary caregivers of critically ill children
- Parents/primary caregivers of children presenting for sexual or physical abuse.
- Parents who do not live in the household with the child.
- Non-Smokers
- Speak a language other than English or Spanish.

## Data Collection

### Patient recruitment

Based on a piloted survey performed with questions generated from a compilation of previous studies done by Dr. Melinda Mahabee -Gittens, the following method was determined to be most effective:

- The data will be collected by research assistants or Dr. Michelle Arzubi. The researchers will receive training regarding the study, the manner in which to approach the parents, to ask questions in a non-judgmental manner, and to collect the data.
- The researchers will approach the parents of the patients once the patients have been triaged, roomed, and seen by either the resident physician, or attending. The researchers will ask the physicians whether or not it is appropriate to see the particular patient’s parents / caregivers.
- Once the researcher is in the room, the conversation will be similar to the following
- “Good morning/ afternoon/ evening, Mr. /Mrs. /Ms. Smith. My name is Dr. Michelle Arzubi. I was wondering whether you would be interested in participating in a research study.” If yes → “This is a study based on smoking. Therefore, my next question is: do you smoke?” If they answer no →“Thank you very much for your time, but at this time, we are only enrolling people who smoke” If yes → “Thank you. You will now be asked approximately 30 questions, which should take no more than 10 minutes of your time. These questions are not meant to judge you or make you feel bad in any way. If at any point in time you feel uncomfortable, please let me know, and we will stop the questions. I have many family members and friends that smoke and understand that smoking is a hard habit to break, both because it’s used as a stress-reliever and it is also addicting. I am handing you a sheet that explains a bit more about this study and why we are conducting it at this time.”
- The questions will be handed to the parents in 8½” x 5½” cards to follow along, while the researcher asks them the questions, and enters the answers on a portable computer. Handing out cards and asking the parents the questions directly, made the parents more comfortable, and clarifies any questions the parents may have.

### Statistical consideration

**Figure 1:**
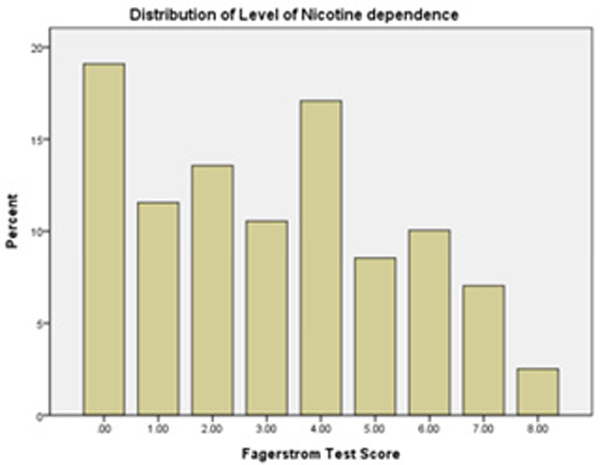
Fagerstrom Test.

**Figure 2:**
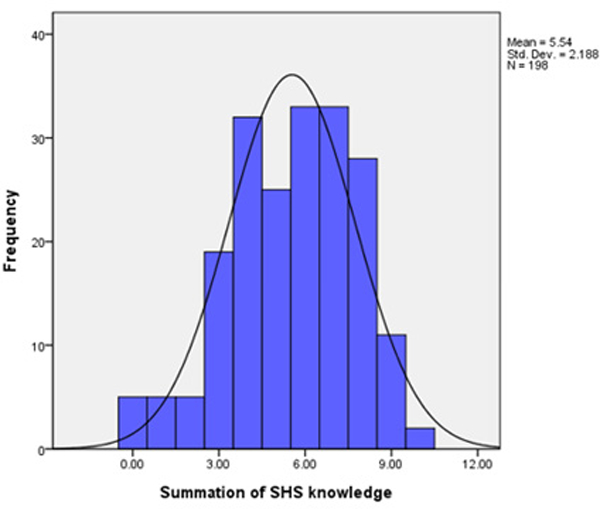
Summation of SHS Knowledge.

**Figure 3:**
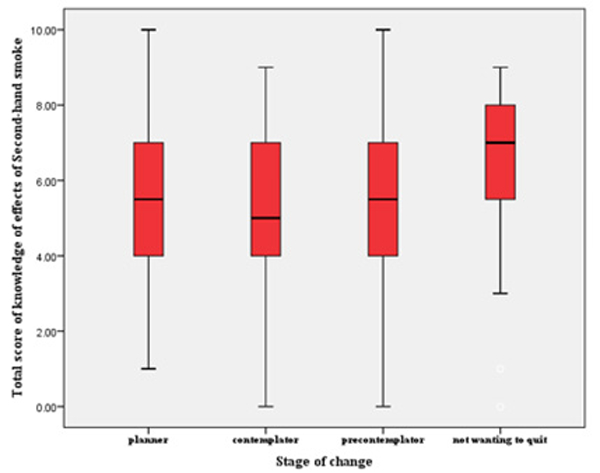


**Figure 4:**
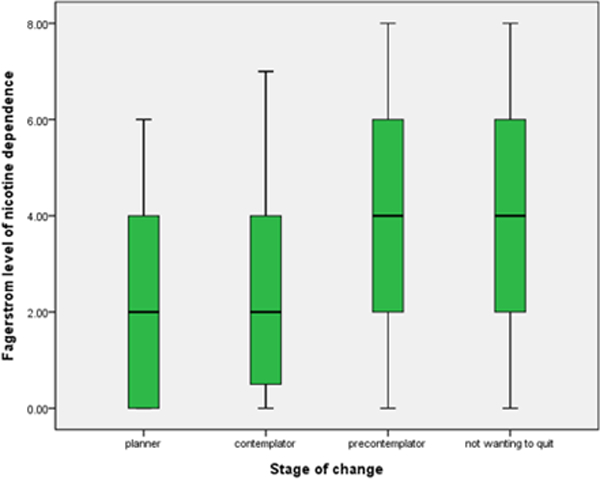


With the assistance of Ashley Sherman, MA, Children’s Mercy statistician, it was determined that with a sample size of 200, there would be a high level of precision for estimating the correlation coefficient. If the correlation is as high as 0.70, the 95% confidence interval for estimating the correlation will range from 0.62 to 0.76. If the correlation is as high as 0.80, the 95% confidence interval for estimating the correlation is calculated to range from 0.74– 0.85 (Figure 1–4).

### Outcome measures

- Level of contemplation/motivation will be defined as: precontemplation → no intention of quitting; contemplation (intend to quit within → the next 6 months; preparation → interested in quitting in the next 30 days and recent attempt.
- Level of parental understanding of the relationship between their smoking and child’s illness will be defined as agreeing or strongly agreeing with the effects of smoking on their child’s health.
- These results will be divided based on the child’s diagnoses. One group will be diagnoses associated with second hand smoke exposure, and the second group will be diagnoses not related to second hand smoke exposure.
- Diagnoses associated with second hand smoke exposure are: Upper respiratory tract infections, Bronchiolitis, Pneumonia, Asthma, Ear infection, difficulty sleeping, or behavior problems.

## Results

A total of 220 parents were asked to participate in the survey, and 200 completed the survey. The 20 parents that did not complete the survey had a variety of reasons for not doing so. The answers varied from lack of desire to participate, to not having enough time. The demographics of the 200 patients are shown in (Table 1–9).

## Figures and Tables

**Table 1:** Description of Basic Demographics in Evaluable Parents who smoke presenting to the Pediatric Emergency Department.

Demographics	Evaluable Parents
**Child’s insurance**	
Commercial	37(18.5%)
Medicaid	148(74%)
None / self-pay	15(7.5%)
**Primary care doctor**	
Yes	86(43%)
No	113(56.5%)
No reply	1(0.5%)
**Race**	
White	96(48%)
Black	85(42.5%)
Other	19(9.5%)
**Education level**	
Less than High School	50(25%)
High School graduate	75(37.5%)
Post High School graduate	75(37.5%)
**Care giver presenting with child**	
Mother	128 (64%)
Father	54(27%)
Other relative	18(9%)
**Child lives with care giver**	
Yes	187(93.5%)
No	13(6.5%)

**Table 2:** Smoking behavior in Evaluable Parents.

Smoking Behavior	Evaluable Parents Answers
**How soon after you wake up do you smoke your first cigarette?**	
< 5 minutes	46(23%)
6 – 30 minutes	60(30%)
31 – 60 minutes	32(16%)
> 1 hour	62(31%)
**Do you find it difficult to refrain from smoking places where it is forbidden?**	
Yes	24(12%)
No	175(87.5%)
**Which cigarette would you hate most to give up?**	
First one in the morning	103(51.5%)
Any other	97(48.5%)
**Do you smoke more frequently during the first hours after awakening than during the rest of the day?**	
Yes	59(29.5%)
No	141 (70.5%)
**Do you smoke even if you are so ill that you are in bed most of the day?**	
Yes	62(31%)
No	138 (69%)
**How many cigarettes do you smoke per day?**	
10 or less	128 (64%)
More than 10	72(36%)

**Table 3:** Fagerstrom level of nicotine addiction.

		Frequency	Percent
Valid	0.00	38	19
	1.00	23	11.5
	2.00	27	13.5
	3.00	21	10.5
	4.00	34	17
	5.00	17	8.5
	6.00	20	10
	7.00	14	7
	8.00	5	2.5
Missing		1	0.5

**Table 4:** Statistics.

Fagerstrom Scale	
Mean	3.1106
Median	3
Std. Deviation	2.34582

**Table 5:** Description of level of contemplation to quit smoking.

Quitting Importance Question	Evaluable Parents Answers
**How important is stopping smoking to you?**	
Not important	24(12%)
Somewhat important	60(30%)
Very important	116(58%)
**Level of contemplation**	
Not wanting to quit	36(18%)
Pre-contemplator	57(28.5%)
Contemplator	65(32.5%)
Planner	42(21%)

**Table 6:** Second hand smoking effects.

Second Hand Smoking Knowledge	Evaluable Parents Answers
**Child with SHS related illness in the last 12 months**	
Yes	179(89.5%)
No	21(10.5%)
**If you keep smoking, how likely is it that the child’s current health would change**	
Health would change	149(74.5%)
Health would not change	49(24.5%)
Missing	2(1%)
**What is the chance your child will develop a serious disease because of the smoking?**	
Low chance of serious disease	125(62.5%)
50% chance of serious disease	50(25%)
High chance of serious disease	23(11.5%)
**SHS effects knowledge: SHS may cause the following**	**Positive answers**
SIDS	96(48%)
Asthma	186 (93%)
Colds / URI	120 (60%)
Pneumonia / Bronchiolitis	170 (85%)
Ear infections	99(49.5%)
Behavioral problems	42(21%)
Sleep disturbance	82(41%)

**Table 7:** Summation scale of SHS knowledge.

Summation score	Frequency	Percent
0	5	2.5
1	5	2.5
2	5	2.5
3	19	9.5
4	32	16
5	25	12.5
6	33	16.5
7	33	16.5
8	28	14
9	11	5.5
10		

Summation of SHS knowledge scale from 0 (low) −10 (high).

**Table 8:** Analysis of level of Nicotine dependence and stage of change.

	Sum of Squares	df	Mean Square	F	Sig.
Between Groups	108.04	3	36.013	7.155	0
Within Groups	981.527	195	5.033		
Total	1089.568	198			

**Table 9:** Nicotine dependence - Fagerström level [7].

Stage of Change	Mean	N	SD
planner	2.3095	42	1.9442
contemplator	2.5	64	2.17489
precontemplator	3.8421	57	2.4985
not wanting to quit	3.9722	36	2.2613
Total	3.1106	199	2.34582
